# Compartment Syndrome of the Hand: Beware of Innocuous Radius Fractures

**Published:** 2014-01-20

**Authors:** Francesco Maria Egro, Matthew Robert Frederick Jaring, Asif Zafar Khan

**Affiliations:** Department of Trauma and Orthopaedics, Frenchay Hospital, Bristol, United Kingdom

**Keywords:** compartment syndrome, complication, hand, radius fracture, injury

## Abstract

**Objective:** Describe an unusual presentation of compartment syndrome of the hand. **Methods:** An unusual and severe case of compartment syndrome of the hand occurred in a 68-year-old woman following an innocuous distal radius fracture and ulnar styloid avulsion fracture. **Results:** The patient required surgical decompression and amputation of the index, middle, ring, and little fingers. **Conclusions:** This article highlights the importance of clinical suspicion toward postinjury compartment syndrome even if a fracture is not adjacent to the compartment itself and no clear vascular disruption is present.

Compartment syndrome is a well-described limb-threatening complication of the leg and forearm, but not as common in the hand. Limb vascularity is affected as the compartment pressure increases beyond the level required to achieve adequate perfusion. It results in ischemia and tissue necrosis, leading to neurologic deficit, muscle necrosis, ischemic contractures, infection, delayed healing of fractures, and possible amputation.

A thorough history and clinical examination are at the basis for this grave diagnosis, but sometimes one is mislead by the apparently harmless type, severity, and site of injury.

This article presents a case of compartment syndrome of the hand as a complication of an innocuous distal radius fracture and ulnar styloid avulsion fracture.

## METHODS

**Figure 1:**
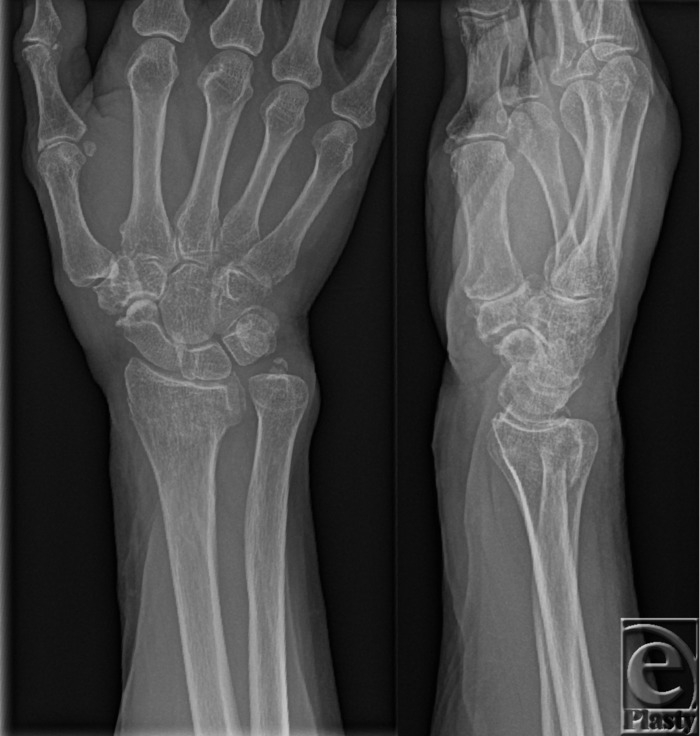
Anteroposterior and lateral radiographs of the right wrist showing a Frykman II displaced distal radius fracture and ulnar styloid avulsion fracture.

A 68-year-old woman presented to the Emergency Department following a fall on the outstretched right arm. She had a background of known hypertension and hypercholesterolemia and her medications included alendronate, simvastatin, lisinopril, and aspirin. The patient was diagnosed radiologically with an extra-articular (Frykman II) displaced distal radius fracture and ulnar styloid avulsion fracture (see Fig 1).

No neurovascular compromise was present at the time. The patient was placed in a dorsal slab without prior manipulation, elevated in a sling, given appropriate analgesia, and advised to return for follow-up to the fracture clinic.

The following day she was seen again in the Emergency Department with increased pain in the right hand. No signs of compartment syndrome were thought to be present at the time and she was sent home with further analgesia and reassurance.

The pain in her right hand worsened overnight and she presented again to the Emergency Department with paresthesia and decreased digital movement the following day.

**Figure 2:**
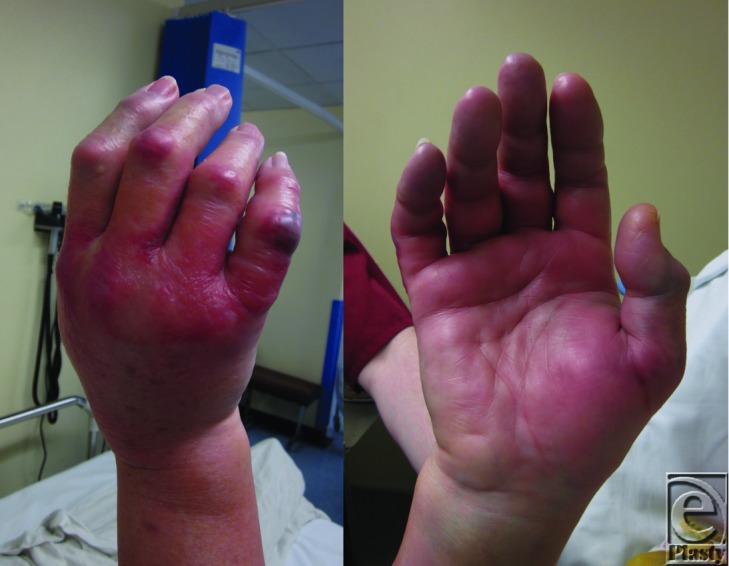
Dorsal and palmar aspect of the hand showing signs of compartment syndrome of the right hand.

On physical examination, the dorsal slab was found to be tight distally and therefore was removed. The forearm muscles were soft and nontender. Marked swelling of the entire right hand and a blue blister in the little finger were observed (see Fig 2). Thenar and hypothenar eminences were soft. The digits were pale and tender and the index, ring, and little fingers appeared mottled. Radial pulse was present; capillary refill was less than 2 seconds in the palm, and 3 to 4 seconds in the index, middle, and ring fingers. Sensorium was obtunded in all fingers except the little finger. Movements were limited with only 15 to 30 degrees at the metacarpophalangeal and interphalangeal joints. Pain was exacerbated on passive flexion.

The patient was diagnosed with compartment syndrome of the right hand secondary to distal radius and ulnar styloid fractures.

## RESULTS

**Figure 3:**
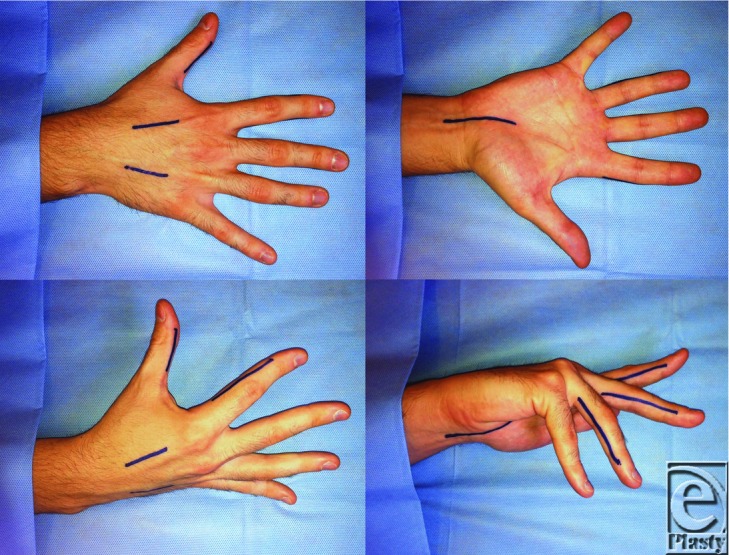
Diagram illustrating the decompressive fasciotomy of the right hand. These include a carpal tunnel incision with extension into forearm, dorsal incisions over second and fourth metacarpals with decompression deep to fascia, and midlateral incisions (radial bands right index finger and ulnar bands right thumb, index, middle, and ring fingers).

The patient's blister was de-roofed and a decompressive fasciotomy of the right hand (see Fig 3) was performed:

– carpal tunnel incision with extension into forearm
– dorsal incisions over second and fourth metacarpals with decompression deep to fascia
– midlateral incisions—radial bands right index finger and ulnar bands right thumb, index, middle, and ring fingers.

Tetanus and 3-day antibiotic prophylaxes were given, and the hand was elevated in a Bradford sling. Two days postoperatively, a CT angiogram was performed showing normal calibre and opacification of the right upper limb arterial supply from axillary artery to the terminal digital arteries.

Day 3 postoperatively, the patient was brought back to theatre for a second look at the fasciotomy wounds. Little and ring finger pulps had fixed staining and no capillary refill on pinprick. Also a small necrotic area in the little finger was noted. Middle and index fingers were fixed stained with poor capillary refill on pinprick. Carpal tunnel, forearm, and thenar eminence incisions were closed. The dorsum of hand was covered with 2 split skin grafts, and the finger incisions were left open.

Three days later, signs of necrosis on tips of the thumb, index, middle, ring, and little fingers were observed.

Four days later, it was decided to amputate middle, ring, and little digits through middle phalanx and index through the proximal interphalangeal joint because of established necrosis dorsally at distal interphalangeal joint level and volarly at proximal interphalangeal joint level.

The patient will be followed up by orthopedic as well as plastic surgery teams and will be provided hand therapy to maximize hand function.

## DISCUSSION

Distal radial fractures are common. They account for approximately 15% of all adult fractures.^1^ These may lead to a series of complications, including compartment syndrome of the forearm with a prevalence of less than 1%.^2^^,^^3^

The incidence of hand compartment syndrome is an even rarer complication with fewer cases described in the literature. When it does occur it is generally associated with a range of more common injuries, including fractures, burns, crush injuries, arterial injuries, limb decompression injuries, and iatrogenic origin.^4^

One study found that 13 of 17 cases of hand compartment syndrome reviewed were believed to be as a result of the administration (either iatrogenically or recreationally) of intravenous or intra-arterial medications or narcotics.^5^

Where fractures were thought to be the causative factor in the diagnosis of compartment syndrome of the hand, these were generally present in the small bones of the hand and associated with crush injuries.^5^

In the few cases of associated ulnar or radial fractures, the type of injury was much more severe, including compression or traumatic crush injuries of the forearm.

A case reported in the literature that did show noncrush fractures leading to the syndrome was due to bony displacement causing arterial rupture, with the latter appearing to be the primary causative pathology.^6^ Vascular integrity was confirmed surgically and radiologically in the presented case and yet irreversible necrosis of the digits occurred.

Another aspect to be considered is the severity of the fracture itself. A study looking at compartment syndromes in children found that in the majority of cases, the fracture was so severe that the use of an external fixator was required.^7^ This is in stark contrast to the presented case, where displacement of fracture did occur, but minimally and within acceptable limits therefore requiring simple splinting by means of a dorsal slab.

When the same study investigated the mechanism of trauma leading to compartment syndrome, it showed the majority of patients had been injured as pedestrians colliding with a vehicle. The fall onto the outstretched arm present in our case involved a significantly lower energy than this.^7^

Importance should be given to the etiology, mechanism, severity, and concomitant injuries since these can all provide a clue on the diagnosis of compartment syndrome of the hand.

All cases reported in the literature highlight the importance of early diagnosis of such a complication. But none provide evidence of the possibility of such a devastating complication following a nonadjacent, displaced, low energy fracture with absent concomitant injuries, requiring only simple splinting and analgesia. This is of importance because this type of fractures is common and is seen on a daily basis in the emergency department by nonhand specialists.

Compartment syndrome should be prevented by avoiding an overly tight cast and by always maintaining a high index of suspicion, especially if the pain is not proportional to the type of injury or the patient seeks medical attention due to worsening pain or change in sensation or color. In the latter cases, the cast should be removed and the hand be examined. If compartment syndrome is diagnosed, early intervention is key for the survival of the digits.

A further interesting point is the fact that despite decompressive treatment and radiological confirmation of vascular patency of the hand, the patient still ended up requiring amputation of the index, middle, ring, and little fingers secondary to necrosis. The need for amputation has been reported in other cases but in clear minority and mainly following intravenous infiltration of drugs^5^—never following such a minor injury as the one presented.

This article highlights the importance of clinical suspicion toward compartment syndrome postinjury even if a fracture is not adjacent to the compartment itself and no clear vascular disruption is present. As shown the sequelae of compartment syndrome can be disastrous to the patient, and given the high incidence of distal radius fractures, we believe this article is even more relevant, describing an initial injury that virtually all junior doctors will encounter during their early training.
